# Sishen Pill Maintained Colonic Mucosal Barrier Integrity to Treat Ulcerative Colitis via Rho/ROCK Signaling Pathway

**DOI:** 10.1155/2021/5536679

**Published:** 2021-12-09

**Authors:** Xiao-Yun Zhang, Hai-Mei Zhao, Yi Liu, Xiu-Yun Lu, Yan-Zhen Li, Qi-Hong Pan, Hai-Yan Wang, Wei Ge, Duan-Yong Liu

**Affiliations:** ^1^Department of Postgraduate, Jiangxi University of Traditional Chinese Medicine, Nanchang 330004, Jiangxi, China; ^2^School of Basic Medical Sciences, Jiangxi University of Traditional Chinese Medicine, Nanchang 330004, Jiangxi, China; ^3^Science and Technology College, Jiangxi University of Traditional Chinese Medicine, Nanchang 330004, Jiangxi, China; ^4^Doctoral Candidate of 2017, Jiangxi University of Traditional Chinese Medicine, Nanchang 330004, Jiangxi, China; ^5^Affiliated Hospital of Jiangxi University of Traditional Chinese Medicine, Nanchang 330006, Jiangxi, China; ^6^Key Laboratory of Pharmacology of Traditional Chinese Medicine in Jiangxi, Nanchang 330004, Jiangxi, China

## Abstract

Sishen Pill (SSP) is a classical prescription of traditional Chinese medicine and often used to treat gastrointestinal diseases, including ulcerative colitis (UC). However, its mechanism is still unclear. We aimed to determine the mechanism of SSP in the treatment of UC by investigating if it maintains the integrity of the intestinal mucosal barrier via the Rho A/Rho kinase (ROCK) signaling pathway. Administration of 2,4,6-trinitrobenzene sulfonic acid (TNBS) successfully induced chronic UC in rats, while the treatment effect of SSP was evaluated by body weight change, colonic length, colonic weight, colonic weight index, histological injury score, and pathological injury score after colitis rats were treated for 7 days. TNF-*α* and IL-1*β* levels were analyzed by ELISA, and the proteins of PI3K/Akt and RhoA/ROCK signaling pathway and junction proteins expression were measured by western blotting assay, and the distribution of Claudin 5 was shown by immunofluorescence. SSP significantly improved the clinical symptoms of colitis in rats and reduced the expression of p-RhoA, ROCK1, PI3K, and Akt in the colon mucosa, while it increased the expression of p-Rac and related proteins (Claudin-5, JAM1, VE-cadherin, and Connexin 43). In addition, SSP increased p-AMPK*α* and PTEN proteins expression, decreased Notch1 level, and hinted that activation of the PI3K/Akt signaling pathway was inhibited. In conclusion, SSP effectively treated chronic colitis induced by TNBS, which may have been achieved by inhibiting PI3K/Akt signal to suppress activation of the Rho/ROCK signaling pathway to finally maintain the integrity of the intestinal mucosal barrier.

## 1. Introduction

Ulcerative colitis (UC) is a nonspecific intestinal inflammatory disease. At present, most treatment methods have failed to reduce the incidence of UC. The specific etiology of UC is unclear but is mainly related to genetic susceptibility, immune response disorders, and epithelial barrier damage [1]. An increasing number of studies have reported that the decline of barrier function in intestinal epithelial cells is positively correlated with the degree of mucosal inflammation in UC patients [2, 3]. Intestinal epithelial intercellular connections are the main components of the intestinal mechanical barrier [4], which is mainly composed of tight junctions (TJs) and the cytoskeleton system. When the intestinal epithelial junction is destroyed or the cytoskeleton is damaged, the mucosal barrier integrity of intestinal epithelial will be destroyed to induce ulcer formation.

Rho A/ROCK plays an important role in maintaining the connection between intestinal epithelial cells. It can regulate reconstruction of the F-actin skeleton. Many studies showed that Rho/ROCK can affect the cytoskeleton and its close connections by inducing cell scaling due to myosin light chain, thus changing the permeability of the intestinal mucosal barrier [5]. Segain et al. found that ROCK inhibitor (Y-27632) improved colitis and reduced intestinal permeability in UC patients and in an animal model of colitis [6]. It is suggested that this may be an effective approach in preventing the attack of chronic UC by interfering with the Rho/ROCK signal to maintain the integrity of the intestinal mucosal barrier.

In recent years, traditional Chinese medicines have been widely used in the treatment of UC [7]. Sishen Pill (SSP) is a classic prescription of traditional Chinese medicine used in the treatment of gastrointestinal diseases including UC. Our previous studies have shown that SSP effectively alleviated colonic colitis induced by 2,4,6-trinitrobenzene sulfonic acid (TNBS), inhibited the expression of inflammatory cytokines, such as tumor necrosis factor (TNF)-*α*, interleukin- (IL-) 17, and IL-1*β* in colonic mucosa, and suppressed excessive apoptosis of colonic epithelial cells [8, 9]. However, whether the effect of SSP treated chronic UC can be achieved by regulating Rho/ROCK signaling has not been determined. Therefore, in the present study, the classic IBD drug 5-aminosalicylic acid (5-ASA) as a positive control drug is used to evaluate the therapeutic effect of SSP [8–10], and we want to assess whether SSP could alleviate TNBS-induced UC by maintaining the integrity of the intestinal mucosal barrier via regulating Rho/ROCK signaling pathway.

## 2. Materials and Methods

### 2.1. Drugs

TNBS (batch number 2508-19-2) was from Sigma (St. Louis, MO, USA) and SSP (batch number 17080051) was obtained from Tongrentang Natural Medicine Co., Ltd. (Tangshan, China), composed of Evodiae Fructus (EF), Psoraleae Fructus (PF), Semen Myristicae (SM), and *Schisandrae chinensis* fructus (SCF). Zhang and colleagues in 2018 analyzed the contents of nine major bioactive components of SSP (batch number 17080051) using high performance liquid chromatography coupled with electrospray tandem mass spectrometry, which contained deoxyschizandrin (72.6 *μ*g/g), *γ*-schizandrin (131.5 *μ*g/g), schizandrin (258.0 *μ*g/g), schizandrol B (71.2 *μ*g/g), schisantherin A (25.1 *μ*g/g), psoralen (131.08 *μ*g/g), isopsoralen (1293.7 *μ*g/g), evodiamine (22.2 *μ*g/g), and rutaecarpine (24.0 *μ*g/g), respectively [11]. Mesalazine enteric-coated tablets (5-ASA) (batch number 170438) were purchased from Sunflower Pharmaceutical Company (Heilongjiang, China).

### 2.2. Animals

Thirty-two male Sprague-Dawley (SD) rats weighing 200 ± 20 g (Animal Certificate No. SCXK (Xiang) 2018-0002) were purchased from Hunan Slake Jing Da Experimental Animal Co., Ltd. (Changsha, China). All animals had free access to the animal center standard diet and tap water under a 12 h dark/light cycle and a temperature of approximately 25°C. The rats were acclimatized for 6 days before experimentation. All procedures were carried out in accordance with the protocol outlined in the “Guidelines for the Nursing and Use of Experimental Animals” of Jiangxi University of Traditional Chinese Medicine. These Guidelines (approval No. JZ 2018-106) were approved by the Institutional Animal Care and Use Committee (IACUC) of Jiangxi University of Traditional Chinese Medicine.

### 2.3. TNBS-Induced Colitis

The rats were randomly divided into four groups: the normal group (normal), the TNBS group (TNBS), the TNBS + SSP group (TNBS + SSP), and the TNBS + mesalazine (5-aminosalicylic acid, 5-ASA) control group (TNBS + 5-ASA), with eight rats in each group. According to previous studies, chronic recurrent UC was induced by TNBS [12, 13]. To ensure an unobstructed enema, the rats were fasted for 12 h and then anesthetized with 3% pentobarbital sodium (0.14 mL·kg^−1^). Except for the normal group, the rats in the remaining groups were injected with 5% w/v 100 mg/kg TNBS (dissolved in 50% ethanol) into the colon about 8 cm from the anus using a plastic hose; the anus was squeezed and the rats were kept in the head down position for 10 min. The above procedure was repeated again on the 8th and 15th day. Rats in the Normal group were injected with an equal volume of physiological saline.

### 2.4. Pharmacological Treatments

**Figure 1:**
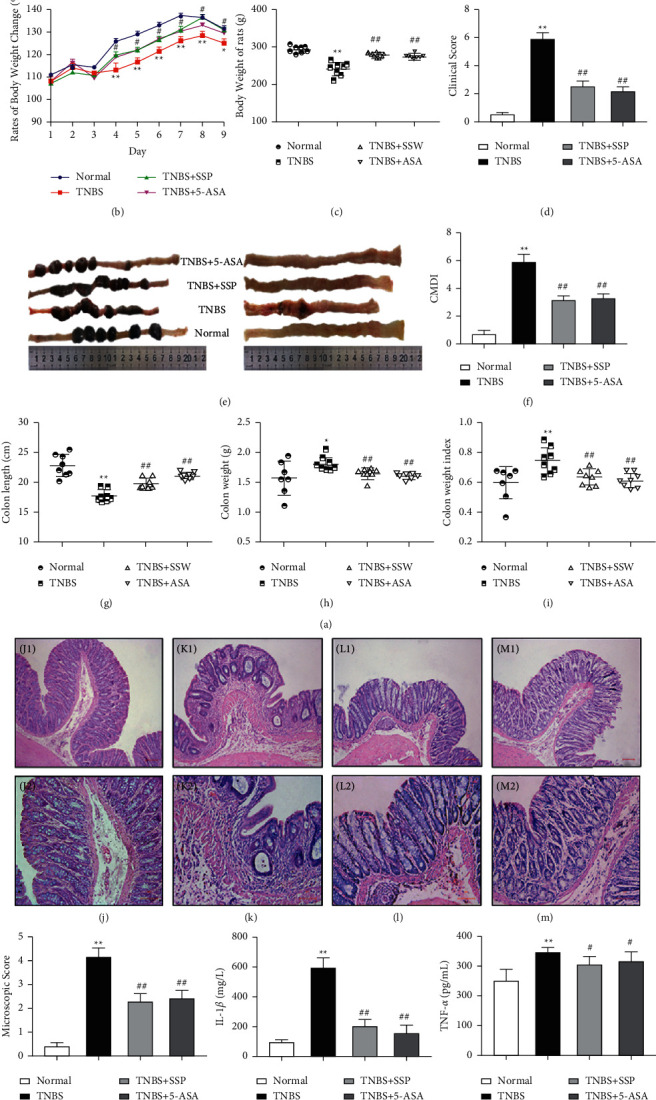
Therapeutic evaluation of SSP-treated chronic colitis in rats. (a) Schedule of Experimental protocol; (b) body weight change during SSP treatment every day; (c) body weight of rats; (d) clinical score; (e) typical images of intact colon and macroscopic view of opened colon; (f) CMDI; (g) colon length; (h) colon weight; (i) colon weight index. Data are presented as means ± SEM (*n* = 8). ^*∗*^*P* < 0.05 and ^∗∗^*P* < 0.01 versus the normal group; ^##^*P* < 0.01 versus the TNBS group. Typical histological images (J, K, L, M) stained with H&E, bar = 100 *μ*m or 200 *μ*m; (j) J1, J2: normal; (k) K1, K2: TNBS; (l) L1, L2 : TNBS + SSP; (m) M1, M2: TNBS + 5-ASA. (n) Microscopic score. (o) IL-1*β*. (p) TNF-*α*. Data are presented as means ± SEM (*n* = 8). ^*∗∗*^*P* < 0.01 versus the normal group; ^#^*P* < 0.05 and ^##^*P* < 0.01 versus the TNBS group.

After 24 h of TNBS enema on the 7^th^ day as shown in Figure 1(a) and according to the body surface area, the rats in the TNBS + SSP group were treated with 5 g·kg^−1^ SSP solution, the rats in the TNBS + 5-ASA group were administered a suspension of 150 mg·kg-1 5-ASA, and the rats in the normal group and the TNBS group were given the same volume of physiological saline. After continuous treatment for 10 days, all rats were sacrificed under pentobarbital sodium anesthesia.

### 2.5. Clinical and Macroscopic Evaluation

Surface body hair, eating, stool traits, body weight, and activity status of rats were observed on a daily basis. The clinical score standard [14] was as follows. The total score was calculated by scoring four items: hunching: 0 no, 1 point yes; wasting: 0 no, 1 point yes; colon thickening: 0, no colon thickening, 1 point, mild thickening, 2 points, moderate thickening, 3 points, extensive thickening; stool consistency: 0, normal beaded stool, 1 point, soft stool, 2 points, diarrhea, 3 points, gross blood. After sacrifice, the colon was rapidly separated and its length was measured, the colon was opened longitudinally along the mesentery, and the feces were removed with phosphate buffered saline. The colon weight index [(colon weight/body weight) x 100%] was then calculated. Colon mucosa injury was scored by the naked eye, and the colonic mucosal damage index (CMDI) scoring standard [15] was used: 0, normal appearance; 1, focal hyperemia, no ulcers; 2, ulceration without hyperemia or bowel wall thickening; 3, ulceration with inflammation at one site; 4, ≥2 sites of ulceration and inflammation; 5, major sites of damage extending >1 cm along the length of the colon; and 6–10, damage extending >2 cm along the length of the colon, and the score was increased by 1 point for each additional 1 cm of damage.

### 2.6. Histological Analysis

A section of colon near the anus was separated and fixed with 4% formaldehyde. The colon tissue was embedded in paraffin, cut into sections (4 *μ*m thick), and then stained with hematoxylin and eosin (H&E). Histology of the colon was observed under an optical microscope. The microscopic pathological injury score [16] consists mainly of inflammatory cell infiltration and tissue injury, and the total score was calculated as follows: (1) the degree of inflammatory cell infiltration was scored as 0 points: no infiltration; 1 point: increased number of inflammatory cells in the lamina propria; 2 points: inflammatory cells extending into the submucosa; 3 points: transmural inflammatory infiltrates; (2) tissue injury degree was scored as 0 point: no mucosal damage; 1 point: discrete epithelial lesions; 2 points: erosions or focal ulcerations; 3 points: severe mucosal damage with extensive ulceration extending into the bowel wall.

### 2.7. Enzyme-Linked Immunosorbent Assay (ELISA)

Colon tissue in the −80°C cryopreservation tube was removed, tissue lysate was added, the tissue was homogenized with an ultrasonic pulverizer, and the coarse lysate was centrifuged for 30 min at 13000 rpm and 4°C. Using an ELISA kit (Thermo Fisher, MA, USA), a portion of the supernatant was removed to measure TNF-*α* (88-7324-22) and IL-1*β* (88-7013-22) levels, and the absorbance was read at 450 nm using an enzyme marker (Bio-Rad, Hemel Hempstead, United Kingdom).

### 2.8. Western Blot Analysis

Protein concentrations of the samples were determined by the BCA microporous plate method. The rat colon tissue samples were homogenized in liquid nitrogen and centrifuged, and the same amount of protein from each sample was separated by SDS-PAGE fractionation and transferred onto polyvinylidene fluoride membranes by the Bio-Rad Western Blot device. The membranes were blocked with 5% bovine serum albumin and probed with the following antibodies at 4°C with shaking for 12 h: Anti-*β*-actin (NO. ab8226) (1 : 3,000), Anti-Rho A (NO. ab187027) (1 : 1,000), Anti-p-Rho A (NO. ab125275) (1 : 1,500), Anti-ROCK1 (NO. ab45171) (1 : 1,000), Anti-Rac1 (NO. ab33186) (1 : 2,000), Anti-Claudin-5 (NO. ab131259) (1 : 1,000), Anti-JAM-1 (NO. ab125886) (1 : 1,000), Anti-Connexin 43 (NO. ab235585) (1 : 500), Anti-VE Cadherin (NO. ab33168) (1 : 500), Anti- Notch1 (NO. ab52627) (1 : 1000), Anti-AMPK*α* (NO. ab32047) (1 : 1,000), Anti-PTEN (NO. ab267787) (1 : 1,000) and Anti-AKT1 (NO. ab233755) (1 : 1,000) (Abcam). The secondary antibodies were horseradish peroxidase coupled anti-mouse or rabbit IgG (1 : 4000 dilution, Abcam). These were incubated with the membranes, and after visualized with an enhanced chemiluminescence (ECL) coloration (Millipore), the film was placed in a gel imager for exposure development. Quantity One software (Bio-Rad) was used to quantify the signal and standardize it as a control, and quantitative data were obtained and analyzed.

### 2.9. Immunofluorescence

For observation of Claudin-5, immunofluorescence staining and confocal microscopy (*n* = 3 for each group) were performed. The tissue sections were treated with 0.01M sodium citrate for antigen retrieval, blocked with 3% normal goat serum at room temperature for 15min, and then incubated with rabbit anti-Claudin-5 antibody (1 : 80, Abcam, Cambridge, UK) overnight at 4°C. After washing, colonic tissues were incubated with a secondary antibody, Dylight 488-labeled goat anti-rabbit IgG (KPL, Gaithersburg, MD, USA) for 2 h at 37°C in the dark. Hoechst 33342 (BD Biosciences Pharmingen, San Jose, CA, USA) was applied to stain nucleus. All sections were photographed under a laser scanning confocal microscope (TCS SP5, Leica, Mannheim, Germany).

### 2.10. Statistical Analysis

All statistical analyses were performed using GraphPad Prism 7.0. The data were expressed as means ± SEM, and the comparison of measurements between groups was determined using one-way ANOVA. *P* < 0.05 was considered statistically significant.

## 3. Results

### 3.1. SSP Improved the Clinical Symptoms of TNBS-Induced Colitis

The TNBS-induced colitis rat model is a commonly used representative animal model for new drug research and development. Clinical observations showed that rats in the TNBS group curled up had decreased appetite, thin stools, and even hematochezia, with decreased body weight. These results were consistent with the clinical score (Figure 1(d)). The CMDI score reflects colonic mucosal injury by the naked eye. The CMDI score in the TNBS group was significantly higher than that in the normal group, while the CMDI scores (Figure 1(f)) in the TNBS + SSP and TNBS + 5-ASA groups were lower than that in the TNBS group. The body weight, colon weight, and colon weight index in rats are important indices and reflect the severity of UC. Compared with the normal group, the weight of rats (Figures 1(b) and 1(c)) in the TNBS group decreased from the 8^th^ day to the 16^th^ day, while the colon weight (Figure 1(i)) and colon weight index (Figure 1(h)) significantly increased. Compared with the TNBS group, body weight (Figures 1(b) and 1(c)), colon weight (Figure 1(i)), and colon weight index (Figure 1(h)) in the TNBS + SSP group and TNBS + 5-ASA group were similar to those in the normal group. Following expansion of the rat colonic wrinkle wall (Figure 1(e)), the TNBS group showed abundant colonic wall thickening, edema, inflammation, and severe intestinal bleeding. The other groups showed smooth colonic wall and no visual bleeding. Shortening of the colon induced by TNBS is the main characteristic of colitis. Compared with the normal group, the colon in rats (Figures 1(e) and 1(g)) in the TNBS group was significantly shorter, and following SSP and 5-ASA treatment, colon length was restored (Figures 1(e) and 1(g)) (*P* < 0.01). These results demonstrated that the symptoms of experimental colitis induced by TNBS were effectively alleviated in the TNBS + SSP group and the TNBS + 5-ASA group.

### 3.2. SSP Improved Colonic Pathological Damage in TNBS-Induced Chronic Colitis

By HE staining and microscopic observations, complete colonic epithelium, ordered arrangement of glands, a few vasodilators, and no inflammatory cell infiltration were found in the normal group (Figure 1(j)). However, pathological changes such as colonic wall thickening, loss of mucosal epithelium, absence of crypt cells, damaged submucosa, abundant inflammatory cell infiltration, and serious submucosal hyperemia and edema were found in the TNBS group (Figure 1(k)). In the TNBS + SSP and TNBS + 5-ASA groups, the colonic epithelium was relatively intact, epithelial repair was obvious, the colonic wall was thickened, a few inflammatory cells were infiltrated, and the hyperemia and edema were surely alleviated (Figures 1(l) and 1(m)). Moreover, the colon microscopic scores in the normal, TNBS + SSP, and TNBS + 5-ASA groups were significantly lower than those in untreated rats with colitis (Figure 1(n)). These pathological results demonstrated that SSP effectively alleviated the colonic mucosal injury induced by TNBS in rats.

### 3.3. SSP Inhibited the Expression of IL-1*β* and TNF-*α* in TNBS-Induced Chronic Colitis

Inflammatory cytokines are important pathological products in IBD pathogenesis. In untreated rats with chronic colitis, the expression of IL-1*β* (Figure 1(o)) and TNF-*α* (Figure 1(p) was markedly increased compared with that in the normal group (*P* < 0.01). IL-1*β* and TNF-*α* expression were markedly decreased following treatment with SSP and 5-ASA (*P* < 0.05 or *P* < 0.01). These results showed that SSP significantly inhibited inflammatory cytokine expression in the colonic mucosa.

### 3.4. SSP Suppressed Activation of Rho/ROCK Signaling in Rats with Colitis

**Figure 2:**
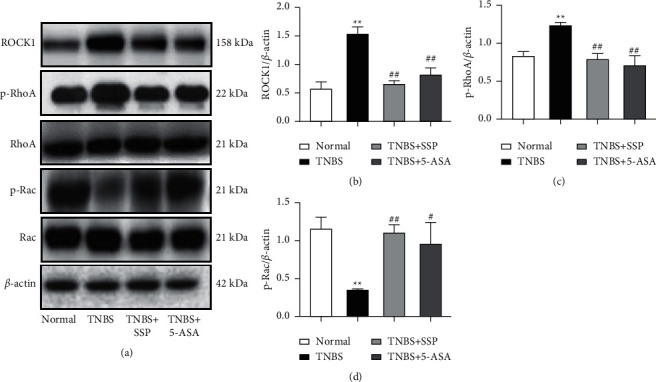
Western blot analysis of the Rho/ROCK signaling pathway. (a) Western blotting of ROCK1, p-Rho A Rho A Rac, and p-Rac. (b) Quantitative analysis of ROCK1. (c) Quantitative analysis of p-Rho A (d) Quantitative analysis of p-Rac. Data are presented as means ± SEM (*n* = 4). ^*∗∗*^*P* < 0.01 versus the normal group; ^#^*P* < 0.05 and ^##^*P* < 0.01 versus the TNBS group.

It has been reported that Rho A/ROCK signal activation can induce IBD. Similarly, in our experiment, compared with the Normal group, p-Rho A and ROCK1 expression was significantly higher in the TNBS group (*P* < 0.01) (Figures 2(a)–2(c)), while in the SSP and 5-ASA treatment group, p-Rho A and ROCK1 expression was significantly reduced compared with the TNBS group (*P* < 0.01) (Figures 2(a)–2(c)). In addition, the expression of p-Rac in the TNBS group was significantly lower than that in the normal group, while the expression of p-Rac in the SSP and 5-ASA-treated group was significantly higher than that in the TNBS group (*P* < 0.05) (Figures 2(a) and 2(d)). These results suggested that SSP inhibited the activation of Rho/ROCK signaling in rats with colitis.

### 3.5. SSP Increased the Expression of Colonic Mucosal Barrier-Related Proteins

**Figure 3:**
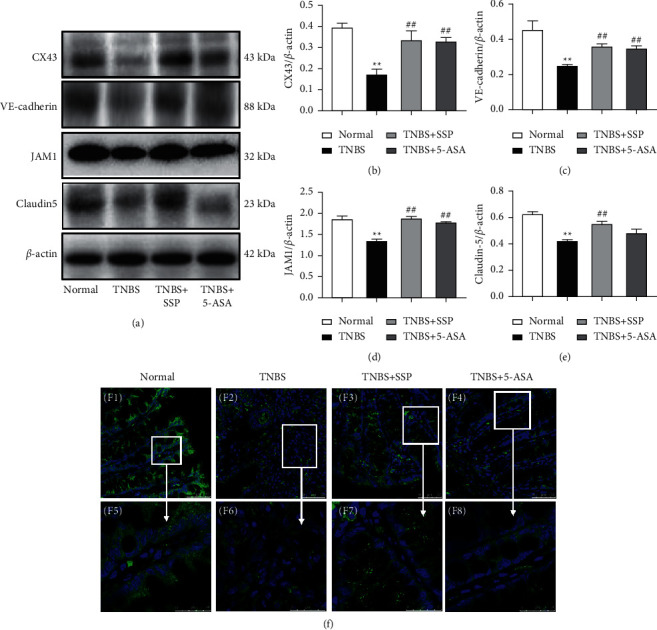
Western blot analysis of key proteins of the colonic mucosal barrier and representative immunofluorescence confocal images. (a) Western blotting of CX43, VE-cadherin, JAM1, and Claudin-5. (b) Quantitative analysis of CX43. (c) Quantitative analysis of VE-cadherin. (d) Quantitative analysis of JAM1. (e) Quantitative analysis of Claudin-5. (f) Representative immunofluorescence confocal images of Claudin-5 in colonic mucosa. The green zone represents the distribution of Claudin-5 and the blue zone nuclei. Bar = 25 *μ*m. Data are presented as means ± SEM (n=4). ^*∗∗*^*P* < 0.01 versus the normal group; ^##^*P* < 0.01 versus the TNBS group.

Western blot analysis showed that the expression levels of Claudin-5, JAM1, CX43, and VE-cadherin (Figure 3) in the TNBS group were significantly lower than those in the normal group. However, after 7 days of treatment with SSP and 5-ASA, compared with the TNBS group, the expression levels of Claudin-5 (Figures 3(a) and 3(e)), JAM1 (Figures 3(a) and 3(d)), VE-cadherin (Figures 3(a) and 3(c)), and CX43 (Figures 3(a) and 3(b)) were significantly higher (*P* < 0.01). However, except for Claudin-5 (Figures 3(a) and 3(e)), the high-expressed JAM1 (Figures 3(a) and 3(d)), CX43 (Figures 3(a) and 3(b)), and VE-cadherin (Figures 3(a) and 3(c)) were found in the TNBS + 5-ASA group (*P* < 0.01) when they were compared with the DSS groups, while confocal microscopy revealed a nearly continuous distribution of Claudin-5 on the surface of colonic epithelium and the junctions of colonic epithelial cells in normal groups. The distribution of Claudin-5 became discontinuous in TNBS group. SSP treatment for 10 days apparently restored the alteration in Claudin-5 distribution caused by TNBS (Figures 3(a), 3(e), and 3(f)). These results showed that SSP increased the expression of colonic mucosal barrier-related proteins to improve the integrity of the intestinal epithelial barrier.

### 3.6. SSP Inhibited Activity of the PI3K/Akt Pathway in TNBS-Induced Chronic Colitis

**Figure 4:**
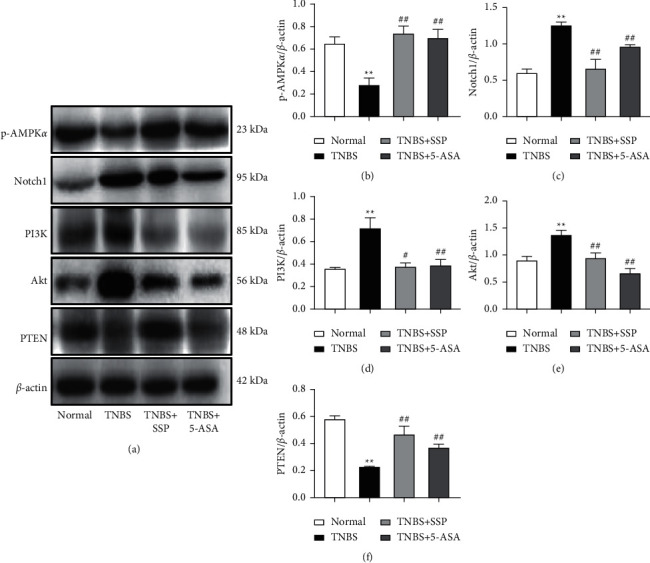
Western blot analysis of the PI3K/Akt signaling pathway. (a) Western blotting of p-AMPK*α*, Notch1, PI3K, Akt, and PTEN. (b) Quantitative analysis of p-AMPK*α*. (c) Quantitative analysis of Notch1. (d) Quantitative analysis of PI3K. (e) Quantitative analysis of Akt. (f) Quantitative analysis of PTEN. Data are presented as means ± SEM (*n* = 4). ^*∗∗*^*P* < 0.01 versus the normal group; ^#^*P* < 0.05 and ^##^*P* < 0.01 versus the TNBS group.

PI3K/Akt signaling is an important upstream pathway of Rho A/ROCK signaling, which plays an important role in regulating Rho A/ROCK signal. Compared with the normal group, the expression of PI3K, Akt and Notch1 in the TNBS group was significantly higher, while the expression of PI3K, Akt, and Notch1 in the TNBS + SSP and TNBS + 5-ASA group was significantly lower than that in the TNBS group (*P* < 0.01 or *P* < 0.05) (Figures 4(a), 4(c)–4(e)). PTEN has protein phosphatase activity, which can dephosphorylate PIP3 and inhibit the activation of the downstream gene Akt. As shown in Figures 4(a) and 4(f), the expression level of PTEN in the TNBS group was significantly lower than that in the normal group, and compared with the TNBS group, the level of PTEN in the TNBS + SSP and TNBS + 5-ASA group was higher. In addition, the expression of p-AMPK*α* (Figures 4(a) and 4(b)) was also increased (*P* < 0.01), suggesting that SSP inhibited activation of the PI3K/Akt signaling pathway in rats with colitis.

## 4. Discussion

The pathological features of UC mainly include mucosal hyperemia, edema, inflammatory cell infiltration, and ulceration. In the present study, these manifestations were present in TNBS-induced chronic recurrent colitis, with shorter colon length, increased colon weight, colon weight index, colon pathological injury score, disorders of colonic mucosa structure, adenoid shedding, massive hyperemia and edema, inflammatory cells infiltration, obvious erosion, and ulcers in the colon. These findings are consistent with previous studies [17–19] and showed that the replication of chronic recurrent colitis induced by TNBS was successful. Following treatment with SSP, the clinical symptoms of colitis were significantly improved, regardless of colon length, colon weight, colon weight index, or pathological injury of the colon mucosa. As a classic drug for inflammatory bowel disease, 5-ASA can inhibit NF-*κ*B to clear oxygen radicals for IBD relief. 5-ASA is a control drug used for therapeutic evaluation in the research and development of new therapeutics for IBD. In the present, 5-ASA has similar protective effect on the TNBS-induced colitis. These results indicated that SSP and 5-ASA effectively attenuated chronic recurrent colitis induced by TNBS.

During the development of this disease, we found that the intestinal mucosal barrier was destroyed. In our experiments, the levels of TJ proteins (Claudin-5, JAM1), GJ protein (CX43), and AJ protein (VE-cadherin) decreased, while the levels of IL-1*β* and TNF-*α* increased, suggesting that the mucosal barrier function in colitis induced by TNBS was destroyed, accompanied with high expression of inflammatory cytokines. It was also found that the Rho/ROCK signaling pathway was activated, the levels of p-RhoA and ROCK1 proteins were increased, and the level of Rac decreased, suggesting that the destruction of intestinal mucosal barrier function caused by Rho/ROCK signal activation plays an important role in the pathogenesis of chronic recurrent colitis induced by TNBS, which is consistent with many other reports [20–24].

**Figure 5:**
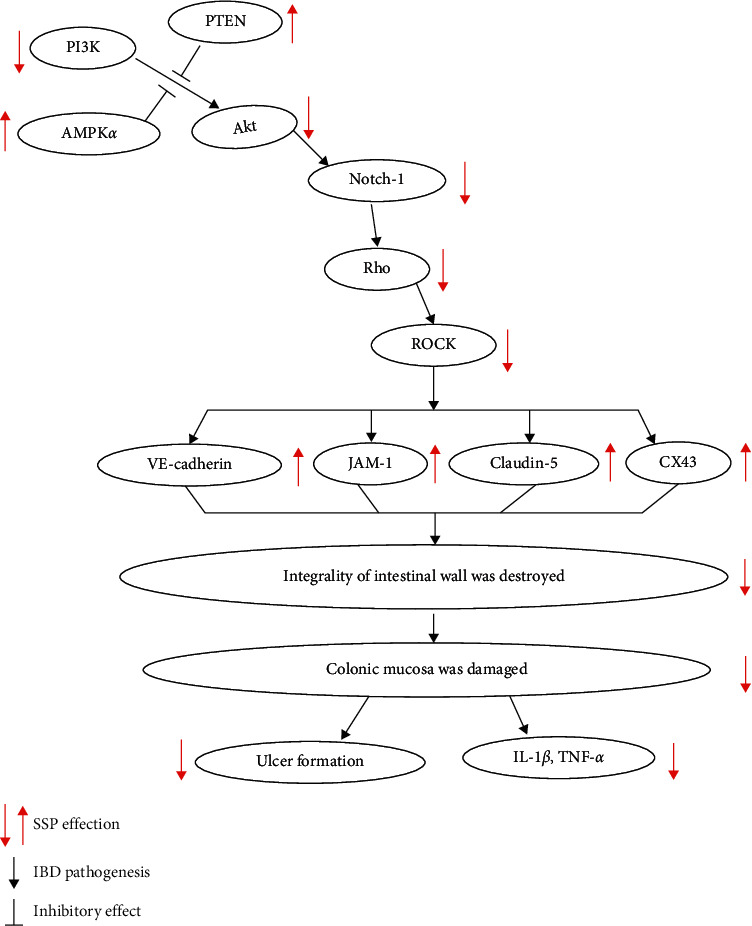
Schematic mechanism of SSP treated mice colitis via PI3K/Akt signaling pathway.

Previous studies have also indicated [25, 26] that the Rho/ROCK signaling pathway plays an important role in maintaining the integrity of intestinal mucosal barrier function and in the pathogenesis of IBD, and the regulation of the signal activation can effectively alleviate colonic mucosal injury in IBD. In our experiments, we found that SSP increased the activity of Rac, inhibited the expression of Rho A and ROCK, increased the expression levels of mucosal-related proteins, such as TJ, GJ, and AJ proteins, and inhibited the inflammatory response. These results indicated that SSP also has an inhibitory effect on the Rho/ROCK signaling pathway. Related research suggested that SSP has pharmacological effects of anti-inflammation, antitumor, antiapoptosis and regulating immunity [27, 28]. Our experiments showed that SSP upregulated the expression of PTEN to inhibit PI3K and Akt, enhanced the expression of p-AMPK*α*, downregulated the expression of Notch1, and inhibited activation of the PI3K/Akt signaling system. According to the literature on the Rho A/ROCK signaling pathway and PI3K/Akt signaling pathway, PI3K inhibitors can inhibit Rho activation, myosin light chain phosphatase (MLCP) activity, and myosin light chain (MLC) phosphorylation [29–31]. Jing and colleagues confirmed that the PI3K/Akt pathway has a regulatory effect on the Rho/ROCK pathway [32]. Thus, it is suggested that the PI3K/Akt signaling pathway is just upstream of the Rho A/ROCK signaling pathway as shown in Figure 5. The PI3K/Akt signal can inhibit the expression of MLCP by promoting ROCK activation, leading to elevate expression of p-MLC. Furthermore, MLC leads to myosin contraction, cell retraction, and rupture of TJ proteins between intestinal epithelial cells. The PI3K/Akt signal inhibited the activation of Rac protein and downstream substance p21-activated kinase (PAK) and then inhibited endothelial and epithelial junctions, ruptured F-actin in the intestine, and damaged the cytoskeleton, resulting in dysfunction of TJ structure and protein expression [5]. The damaged colonic mucosal barrier is a usual pathological character of IBD. There are three kinds of junction complexes at the top of the lateral membrane of the adjacent cells between epithelial cells, namely, tight junctions (TJ) (such as occludin, claudin, JAM, and zonula occludens (ZO)), adhesive junctions (AJ) (such as VE-cadherin and E-cadherin), gap junctions (GJ) ((Connexin43, CX43)), and desmosome junction (DJ). Occludin, claudin, JAM-1, and ZO in colonic epithelial cells play essential roles in intestinal tight junction (TJ) function and are responsible for maintaining cell polarity and related to extracellular permeability [33]. TJ and AJ proteins may regulate junctional permeability by modulating GTP binding to and/or GTP hydrolysis of Cdc42, Rac, and RhoA, while many previous studies had indicated that the expression and/or localization of TJ (including occludin, JAM-1, ZO-1, claudin-3, and claudin-5) was decreased in the inflammatory colon of patients with IBD. In summary, the TJ protein family plays a crucial role in IBD pathogenesis [34, 35]. Simultaneously, the integrality of the intestinal mucosa was destroyed, and then intercellular permeability was increased, a variety of bacteria, endotoxins, and macromolecular substances entered the lower layer of intestinal mucosa and caused destruction of the entire mucosal barrier function. This led to intestinal mucosa damage, congestion, edema, the formation of necrotic substances, the release of inflammatory media, and increased cytokine expression.

By document analysis, we found that the main active constituents of SSP (such as water extract of Evodiae Fructus (EF) and *Schisandra chinensis* polysaccharides) have a great better antidiarrheal, antitransit, and anti-inflammatory by strengthening the mucosal barrier integrity [36, 37]. However, its mechanism is still kept ambiguous. In the present study, SSP inhibited the activation of PI3K/Akt by increasing PTEN expression to suppress activation of the Rho/ROCK pathway and then increased the expression of TJ proteins and maintained the integrity of the colonic mucosal barrier. Pathogenic factors cannot enter the lower layer of intestinal mucosa. As a commonly used drug, SSP is composed of many active components, such as evodiamine, schizandrin A, myrislignan, and macelignan, which have a wide range of pharmacological effects, including anti-inflammation, antitumor, anti-free radicals, and regulating immunity [38–40]. Numerous studies have shown that myrislignan protects skin keratinocytes from ultraviolet B-induced damage and reduces the activation of MAPK and PI3K/Akt [41]. Evodiamine can inhibit cell proliferation by inhibiting PI3K/Akt signal to induce apoptosis and activate MAPK in pleomorphic glioblastomas [42, 43] and effectively inhibit the protein expression of Rho, ROCK1, and ROCK2. In the present study, we thought, as a whole prescription, the effective components of SSP may potentially have a synergistic effect to inhibit PI3K/Akt activation to cause a cascade protective effect by the Rho A/ROCK signal. SSP effectively controlled the levels of Rho A/ROCK signal activation, maintained the balance between Rho A and Rac, and increased junction protein expression to improve the function of the colonic mucosal barrier. SSP also successfully alleviated the pathological colonic injury induced by TNBS. However, which active components of SSP produced inhibitory effects on the Rho/ROCK or PI3K/Akt signaling pathway and their effective targets need to be verified by further investigation.

## 5. Conclusions

Our results demonstrated that SSP effectively treated chronic recurrent colitis induced by TNBS, which may have been achieved by inhibiting PI3K/Akt signal to suppress activation of the Rho/ROCK signaling pathway to finally maintain the integrity of the intestinal mucosal barrier. In the future, we will measure the mitochondrial stress, mitochondrial substrate preference, glycolysis stress, and real-time ATP production rate in colonic tissues by Agilent Seahorse XF and analyze ATPase gene expression by metagenomics to find the correlation between energy metabolism, ATP level and AMPK. Furthermore, we will verify these key proteins or gene activation by RT-PCR or western blotting assay. Meanwhile, we will quantify the MLCK, p-MLCK, ATPase, and F-actin expression by immunofluorescence or WB. We thought that it will be very valuable to explore the mechanism of SSP maintained integrity of colonic mucosa.

## Data Availability

All the data are given in the present manuscript.

## Abbreviations

SSP: Sishen Pill
IBD: Inflammatory bowel disease
UC: Ulcerative colitis
ROCK: Rho kinase
TNBS: 2,4,6-trinitrobenzene sulfonic acid
TJs: Tight junctions
AJ: Adhesion junction
TNF: Tumor necrosis factor
IL: Interleukin.
